# Proton Minibeam Radiation Therapy and Arc Therapy: Proof of Concept of a Winning Alliance

**DOI:** 10.3390/cancers14010116

**Published:** 2021-12-27

**Authors:** Ramon Ortiz, Ludovic De Marzi, Yolanda Prezado

**Affiliations:** 1 Institut Curie, Université PSL, CNRS UMR3347, Inserm U1021, Signalisation Radiobiologie et Cancer, 91400 Orsay, France; Yolanda.prezado@curie.fr; 2 Université Paris-Saclay, CNRS UMR3347, Inserm U1021, Signalisation Radiobiologie et Cancer, 91400 Orsay, France; 3 Centre de Protonthérapie d’Orsay, Radiation Oncology Department, Campus Universitaire, Institut Curie, PSL Research University, 91898 Orsay, France; ludovic.demarzi@curie.fr; 4 Institut Curie, Campus Universitaire, PSL Research University, University Paris Saclay, INSERM LITO, 91898 Orsay, France

**Keywords:** proton minibeam radiation therapy, proton arc therapy, normal tissue dose toxicity

## Abstract

**Simple Summary:**

Normal tissue’s morbidity continues to limit the increase in the therapeutic index in radiation therapy. This study explores the potential advantages of combining proton arc therapy and proton minibeam radiation therapy, which have already individually shown a significant normal tissue’s sparing. This alliance aims to integrate the benefits of those techniques in a single approach.

**Abstract:**

(1) Background: Proton Arc Therapy and Proton Minibeam Radiation Therapy are two novel therapeutic approaches with the potential to lower the normal tissue complication probability, widening the therapeutic window for radioresistant tumors. While the benefits of both modalities have been individually evaluated, their combination and its potential advantages are being assessed in this proof-of-concept study for the first time. (2) Methods: Monte Carlo simulations were employed to evaluate the dose and LET distributions in brain tumor irradiations. (3) Results: a net reduction in the dose to normal tissues (up to 90%), and the preservation of the spatial fractionation of the dose were achieved for all configurations evaluated. Additionally, Proton Minibeam Arc Therapy (pMBAT) reduces the volumes exposed to high-dose and high-LET values at expense of increased low-dose and intermediate-LET values. (4) Conclusions: pMBAT enhances the individual benefits of proton minibeams while keeping those of conventional proton arc therapy. These results might facilitate the path towards patients’ treatments since lower peak doses in normal tissues would be needed than in the case of a single array of proton minibeams.

## 1. Introduction

In recent decades, radiation therapy (RT) has profited from numerous technological advances allowing a very high dose conformation to the tumor along with significant dose reduction to surrounding normal tissues. Examples include techniques such as Intensity Modulated Radiation Therapy (IMRT) [1] or Volumetric Modulated Arc Therapy (VMAT) [2]. Nevertheless, normal tissue toxicities remain the main limiting factor in RT, especially compromising the treatment of radioresistant tumors (i.e., GBM), pediatric cancers or tumors close to highly radiosensitive organs, such as the spinal cord.

Charged particles, such as protons, further increase the tissue sparing-potential with respect to X-rays thanks to their more favorable depth dose deposition [3]. Despite general reduced toxicity as compared with X-rays, proton therapy may also result in severe side effects, such as radiation necrosis [4].

To diminish the side effects and achieve a high therapeutic index for current difficult-to-treat cases, new approaches have been proposed. This is the case of Proton Arc Therapy (PAT) [5] and Proton Minibeam Radiation Therapy (pMBRT) [6].

PAT is considered one of the future promising evolutions in proton therapy dose delivery [5]. Proton arc beams take advantage of the rotating arc beam to diffuse the proton range uncertainty distal and proximal regions and reduce its impact on the final dose distribution. Thereby, PAT has the potential to further improve the quality and robustness of the treatment by enhancing the dose conformity at the tumor level while reducing the total dose received by the patient, optimizing linear energy transfer (LET) distribution, and it may further improve the adoption of dose escalation and hypofractionation [7]. The technique has demonstrated potential clinical benefits in several disease sites or indications [5,8,9,10,11,12]. One of the best current indications for PAT appears to be brain tumors since they usually require short proton ranges and the symmetry of the head provides a greater region over which to arc the beam [13]. Thereby single arcs could replace coplanar arcs, enabling the use of less degraded beams.

Another approach to diminish normal tissue’s toxicity is proton minibeam radiation therapy (pMBRT) [6]. pMBRT is a spatially fractionated RT technique, which uses 0.5–1 mm-width planar beams spaced by 2 to 4 mm. This results in a high spatial modulation of the dose, with areas receiving very high doses (peaks) corresponding to the primary beam paths and areas of low doses (valleys), filled in with the scattered radiation [14]. pMBRT already made proof of a net reduction in normal tissue toxicity as compared to broad beam conventional irradiations [15,16,17,18], while the same or superior tumor control was achieved [19,20,21]. Thus far, only irradiations using one sole array of proton minibeams or two orthogonal arrays in crossfire geometry have been used.

This work highlights the potential advantages of combining these two approaches, PAT and pMBRT, in order to improve the treatment of radioresistant tumors, as these techniques can be very complementary (preservation of healthy tissue with pMBRT, and biological optimisation in the tumor with PAT). This combined delivery method will be referred to as Proton Minibeam Arc Therapy (pMBAT) hereafter. In this work, the potential increase in normal tissue sparing of pMBAT while maintaining the characteristic pattern of peaks and valleys of pMBRT was evaluated. In addition, the impact of the several main parameters in the treatment planning process on the possible reduction in dose to normal tissues of pMBAT was assessed. To the best of our knowledge, this is the first evaluation of pMBAT.

## 2. Materials and Methods

### 2.1. Simulation Geometry and Details

Dose distributions of all treatment cases were computed by means of Monte Carlo (MC) simulations. The simulation code TOPAS (TOolkit for Particle Simulation v3.5 based on Geant4.10.7) was employed [22]. The pencil beam scanning beamline (from a 235 MeV cyclotron) and nozzle of the Orsay proton therapy center (ICPO) were modelled as described in [23].

The physics list, which defines the physical processes involved in the simulation [22], was built using the Geant4_Modular option with the modules recommended for proton therapy (“g4em-standard_opt3” “g4h-phy_QGSP_BIC” “g4decay” “g4ion-binarycascade” “g4h-elastic_HP” “g4stopping” “g4radioactivedecay”) [24,25] as in previous similar pMBRT dose calculations [26]. The range cut for all particles (i.e., production threshold for secondary particles) was set at 0.01 mm. The global uncertainty, calculated as the average statistical uncertainty of voxels with a dose higher than 90% of the maximum dose, was below 3%, as considered in previous studies on pMBRT [26]. Our pMBRT simulations had been previously calibrated and benchmarked against experimental data in water phantoms [27].

**Figure 1:**
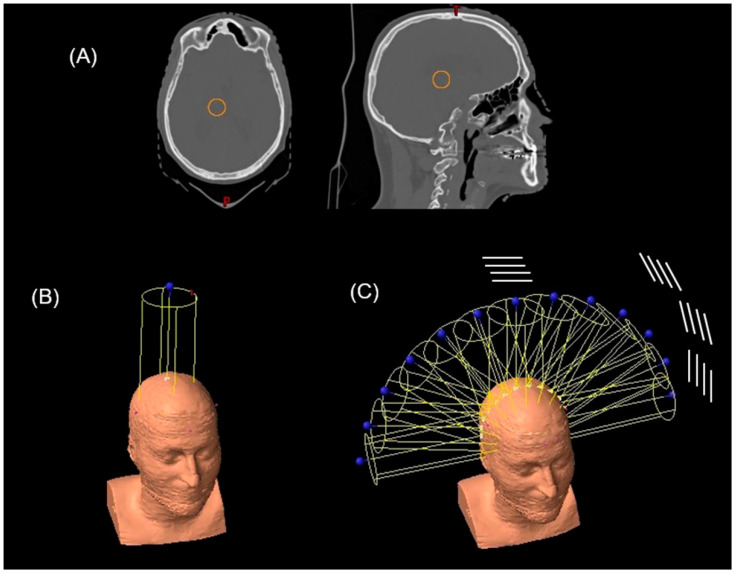
(**A**) PTV (orange solid line) in the axial and sagittal view of the patient CT. Beam arrangement in the (**B**) standard pMBRT (single array) and (**C**) PAT and pMBAT (multi-array) plans. In (**C**), a schematic sketch of the rotation of the slits is represented.

The calculations were performed using fully anonymized computer tomography images of a patient from the ICPO patient database (see Figure 1). All procedures involving patient data were in accordance with the ethical standards, guidelines and regulations of the Institut Curie ethics committee and with the 1964 Helsinki declaration and its later amendments or comparable ethical standards (approval number DATA190299). Since gathered patient data was retrospective and did not directly involve the human participants during this theoretical work, informed consent was not applicable to this study.

### 2.2. Treatment Plans

We compared three types of treatment plans: (i) a single-array pMBRT treatment; (ii) different pMBAT treatments, and (iii) a PAT plan. At the same average dose to the tumor, the reduction in normal tissue doses (peak and valley doses), and maintenance of the spatial modulation of the dose in the normal tissues were considered as figures of merit. To evaluate the possible dose reduction in pMBAT with respect to pMBRT and PAT, depth dose curves both in the peak and valley regions and dose volume histograms (DVH) were computed. To assess the preservation of the spatial fractionation, the peak-to-valley dose ratio (PVDR) in the pMBAT and pMBRT scenarios was also computed. Finally, LET distributions were studied to give an overview of the possibilities of biological optimization with these techniques.

In order to evaluate the dosimetric parameters of our irradiation types, a virtual 2 cm-diameter planning target volume (PTV) was positioned in the centre of the patient’s brain, as in previous works [28,29].

The spot configuration of all the treatment plans presented hereafter was created by the ECLIPSE treatment planning system (Varian Medical Systems, Palo Alto, CA, USA) using the Nonlinear Universal Proton Optimized algorithm (NUPO) (v.15.6.05) with pencil beam scanning algorithm and delivery.

#### 2.2.1. Single-Array pMBRT Treatment

To simulate the single-array pMBRT irradiations, a 65 mm-thick multi-slit brass collimator, optimized for proton minibeam generation [27], was utilized. The collimator was composed of 15 slits separated by a center-to-center (c-t-c) distance of 4, 2.8 or 2 mm for the three cases studied, respectively. The dimension of the slits was 400 μm × 5.6 cm. The irradiation field area was optimized using ECLIPSE and it depends on the array angle. The distance from the collimator exit to the patient’s skin was 5 cm and a 30 mm block of PMMA was placed before the collimator acting as a range shifter as could be done in clinical routine.

#### 2.2.2. PAT Treatments

To simulate the PAT treatments, an arc was made up of a discrete number of fields separated by a constant angle. The PAT plan was composed of up to 13 equally spaced fields forming a 180° arc around the parietal region of the patient’s head (see Figure 1).

#### 2.2.3. pMBAT Treatments

To create the pMBAT treatment, a collimator as previously described was added to the PAT plan detailed in the previous section. Thus, the pMBAT plan was composed of up to 13 equally spaced minibeam arrays. To preserve the spatial fractionation along all of the arc, the rotation of the gantry was carried out around the axis perpendicular to the slits. Figure 1 shows a schematic representation of the collimator slits and their rotation around the patient.

The influence of different parameters (angle between consecutive arrays, number of arrays, centre-to-centre (c-t-c) distances and PTV size) on the pMBAT dose distributions was evaluated. Table 1 reports the different parameters employed in the different configurations studied.

## 3. Results

In this section, the comparative evaluation of the doses deposited on both normal tissues and tumor by pMBRT, PAT and pMBAT, as well as on the influence of the different parameters on the performance of pMBAT, are reported. In addition, we present a comparison of the LET distributions between pMBRT and pMBAT. If not otherwise stated, the pMBAT calculations correspond to case 1 (13 angles, 15°, 4000 μm c-t-c).

### 3.1. Preservation of the Spatial Modulation of the Dose

**Figure 2:**
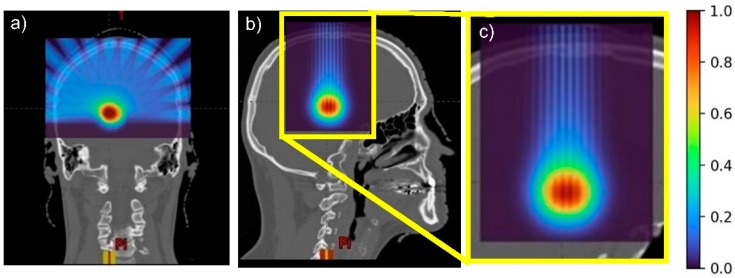
(**a**) Coronal, (**b**) sagittal views, and (**c**) sagittal view zoomed in of the pMBAT dose distributions.

**Figure 3:**
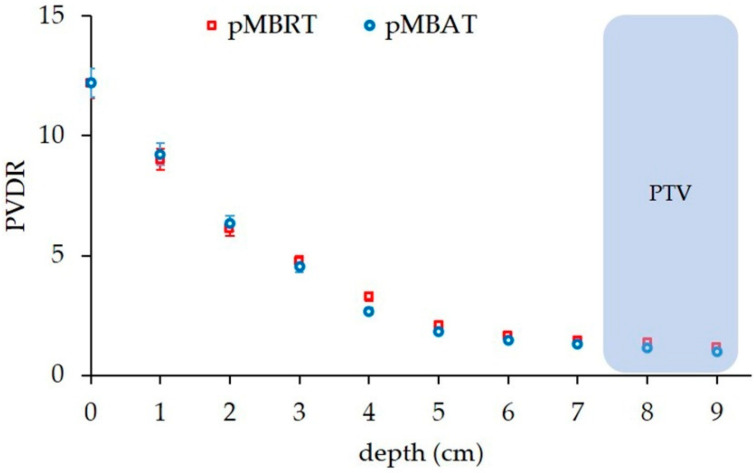
PVDRs at different depths along the beam path in the standard pMBRT (single array) and pMBAT (multi-array) scenarios.

Figure 2 and Figure 3 illustrate the preservation of the spatial fractionation of the dose in pMBAT: neither the pattern of peaks and valleys nor the peak-to-valley dose ratio (PVDR) values are modified in normal tissues by the addition of arrays to form the arc, as compared with a single-array pMBRT treatment. Figure 3 shows the evolution of the PVDR in normal tissues as a function of depth. PVDR values can be found in Supplementary Materials Table S1. In the pMBAT treatment, a quasi-homogeneous dose distribution is achieved in the tumor with a PVDR ranging from 1.06 ± 0.05 to 1.27 ± 0.06. PVDRs were computed as the ratio between peak and valley doses at the center of the lateral profiles.

### 3.2. Dose Reduction to Healthy Tissues

**Figure 4:**
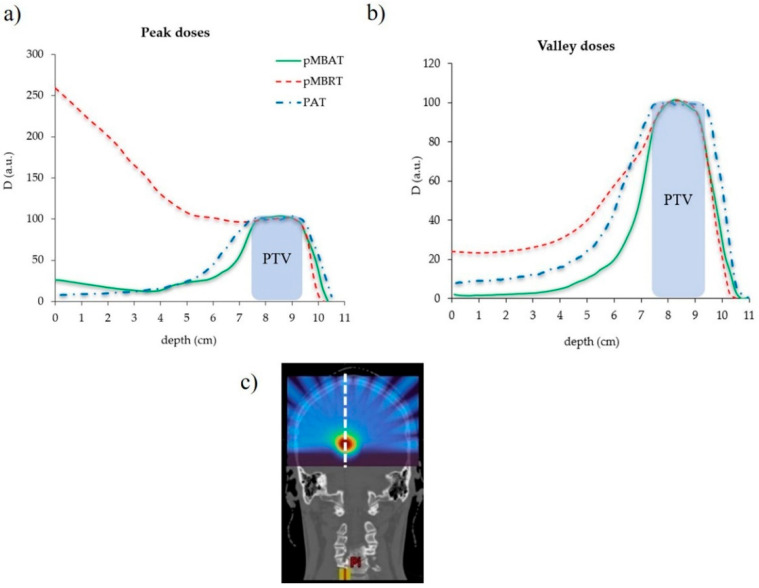
Depth dose profiles at (**a**) peak and (**b**) valleys positions of the standard pMBRT (single array), PAT and pMBAT (multi-array) scenarios. The profile considered is represented by a dotted white line in (**c**). Doses are normalized to the maximum dose at peak and valleys, respectively.

**Figure 5:**
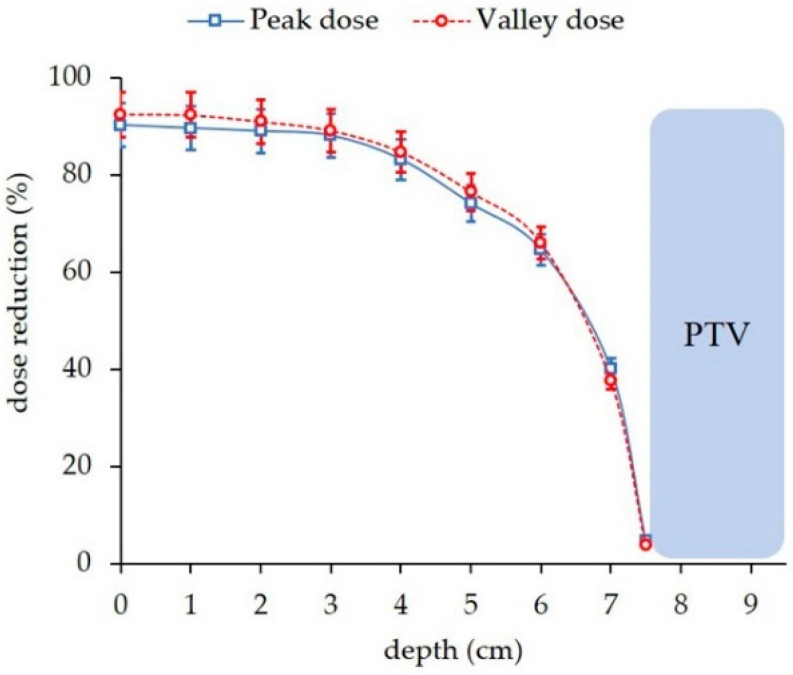
Dose reduction to normal tissue along the beam axis at peak and valley positions in the pMBAT scenario with respect to the standard pMBRT case. The profile considered to evaluate the dose reduction is represented by a white dotted line in Figure 4c.

Figure 4 compares the depth dose curves for the single array-pMBRT, pMBAT and PAT irradiations in the longitudinal direction. pMBAT results in a significant reduction in peak and valley doses to normal tissues as compared to single-array pMBRT. Peak and valley doses are reduced by 90% at shallow depths and by 5% at few millimeters close to the PTV, for the reference case (case 1). Figure 5 shows the evolution of the dose reduction as a function of depth for both peak and valley doses. Similar results were found for all the arrays composing the arc in the pMBAT scenario. These results can be found in Supplementary Materials Figure S1. Peak doses in the pMBAT case are larger at shallow depths and smaller within a few centimeters close to the PTV as compared with PAT. Valley doses to normal tissues in pMBAT are significantly lower than in PAT.

**Figure 6:**
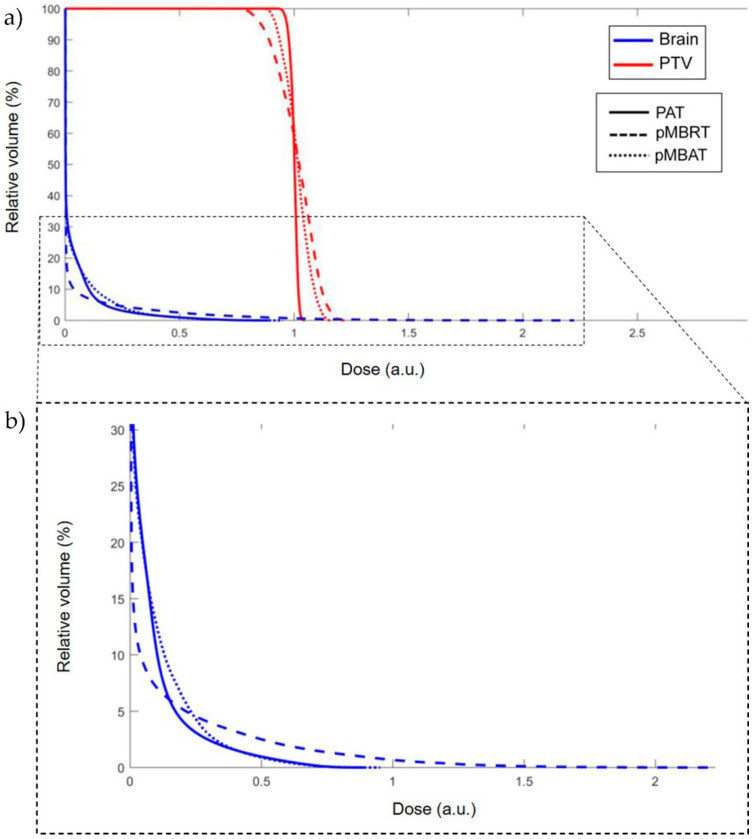
(**a**) DVH for pMBRT, PAT and pMBAT treatments. (**b**) Zoomed in view of (**a**). Doses are normalized to the same average dose to the PTV.

Figure 6 presents the dose-volume histogram (DVH) for the pMBRT, PAT and pMBAT treatments. The PTV was considered as the target volume and the whole brain, excluding the PTV, as the normal tissue. For the sake of comparing seamless and minibeam dose distributions, the dose was normalized to the average dose to the PTV, as considered in pMBRT pre-clinical trials [20,21]. The less steep curve for the PTV in pMBAT and pMBRT with respect to PAT is a consequence of the spatial modulation of the dose in the target. However, tumor control is expected to be maintained or increased as observed in our preclinical experiments [21]. DVH for the normal tissue volume shows a decrease in the high-dose area in the pMBAT case, while a larger volume is covered by low doses, as compared to pMBRT. Integral doses to the healthy tissues were found to be equivalent within their statistical uncertainty in all cases (see Table 2). Then, the decrease in peak and valley doses in pMBAT is achieved at expenses of the irradiation of a larger volume with lower doses.

### 3.3. LET Distributions

**Figure 7:**
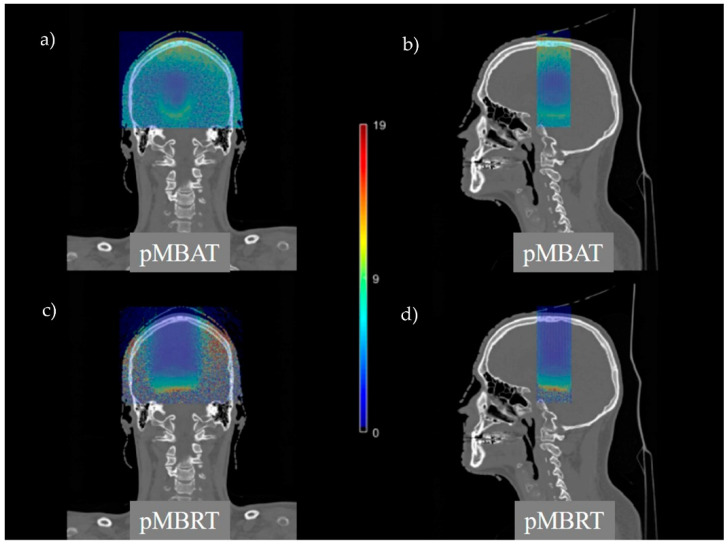
Coronal and sagittal views of LET_d_ distributions in pMBAT (**a**,**b**) and pMBRT (**c**,**d**).

Figure 7 compares LET_d_ distributions of pMBRT and pMBAT treatments. pMBAT reduces the hot spots and the normal tissue volumes receiving high LET values at expenses of a higher volume receiving intermediate-LET values, as compared to pMBRT. Mean LET_d_ values in the PTV and the normal tissue are similar in the three scenarios within their statistical uncertainty (see Table 3).

### 3.4. Influence of the Plan Parameters in the Dose Reduction in pMBAT

The impact of the number of arrays, separation angle between the arrays, c-t-c distance and PTV size on the dose reduction in pMBAT was evaluated.

#### 3.4.1. Influence of the Number of Arrays on the Dose Reduction

**Figure 8:**
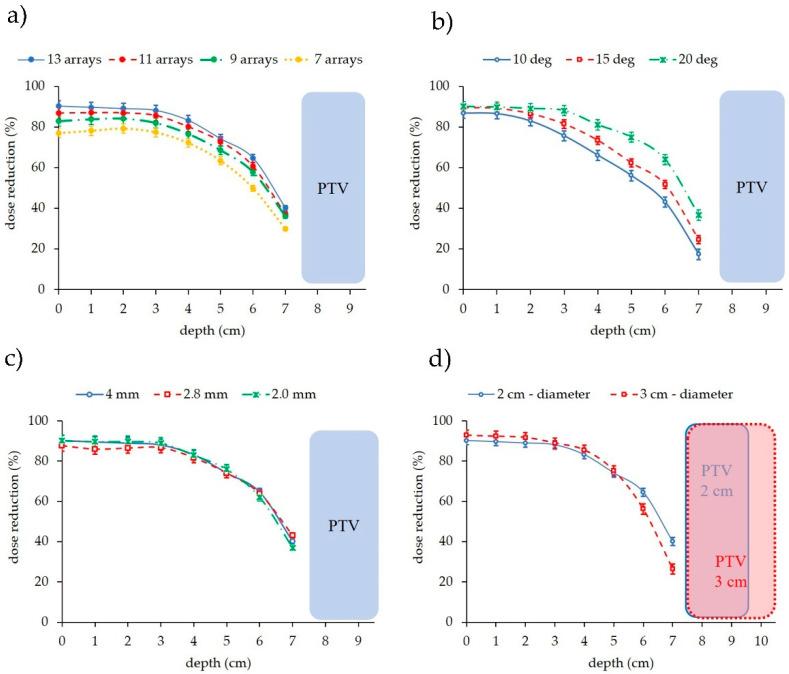
Dose reduction to normal tissues of pMBAT with respect to one-array pMBRT as a function of depth and (**a**) number of arrays forming the arc, (**b**) separation between arrays, (**c**) c-t-c distance, and (**d**) PTV diameter in pMBAT.

Figure 8 depicts the amount of dose reduction to normal tissues with respect to one-array pMBRT as a function of the number of arrays forming the arc. pMBAT plans 1, 2, 3 and 4 were compared. As expected, the greater the number of arrays, the lower the dose to normal tissues. A 15% difference is for example observed between 7 and 13 arrays at shallow depths. The coronal view of dose distribution of the different scenarios where the number of arrays is modified can be found in Supplementary Materials Figure S2.

#### 3.4.2. Influence of the Angular Separation on the Dose Reduction

Regarding the separation between arrays forming the arc, the smaller the angle between the arrays, the larger the overlap of dose at shallow depths. pMBAT plans 5, 6 and 7 were evaluated. The dose to normal tissues at peak and valley regions is reduced by 7% for each 5°-increase on the array spacing on average along the proton range, as illustrated in Figure 8. That difference is higher after the depth at which two different arrays overlap. This overlap can be observed in Supplementary Materials Figure S3, which displays the coronal view of the dose distributions resulting from the treatment plans considering different angular separations. In the case studied, differences in the dose reduction are observed from shallow depths in the case of 10° array separation, with respect to the two other cases, since the arrays overlap close to the patient surface, contrarily to the 15° and 20° cases. Differences between the 15° and 20° array separation plans are observed from ~3 cm depth, where the arrays separated by 15°overlap.

#### 3.4.3. Centre-To-Centre Distance

The c-t-c distance does not affect the dose reduction to healthy tissues. pMBAT plans 8, 9 and 10 were compared. Figure 8 presents the dose reduction in the pMBAT cases utilizing a collimator consisting of slits separated by 4000, 2800 and 2000 μm with respect to the single-array plan using the same respective c-t-c distance. No significant difference was observed between the dose reduction in the three scenarios. Then, the choice of the c-t-c distance does not affect the beneficial dose reduction in pMBAT with respect to standard pMBRT.

#### 3.4.4. PTV Size

The reduction in the dose in pMBAT is dependent on the PTV size. pMBAT plans 11 and 12 were evaluated. The larger the PTV, the lower the dose reduction, as Figure 8 illustrates. As the PTV diameter increases, the extent, i.e., the area, of the planar minibeam is larger to cover the increased extension of the PTV. Consequently, two arrays of the arc separated by the same distance will overlap at shallower depths if the planar minibeam is larger, resulting in a decreased dose reduction with respect to the single-array treatment.

## 4. Discussion

Despite significant improvements in dose conformality in recent decades, normal tissues toxicity remains the main barrier to achieve effective treatments in radioresistant tumors and tumors close to radiosensitive organs. Two recent innovative RT techniques, PAT and pMBRT, have already individually shown a significant potential for morbidity reduction. The aim of this study was to investigate the feasibility and potential dosimetric advantages of the combination of these two techniques into a new approach called Proton Minibeam Arc Radiation Therapy (pMBAT). To the best of our knowledge, this is the first evaluation of combining proton minibeams with PAT, a promising proton therapy technique.

The potential for integrating PAT into the clinic is currently very high. Indeed, the first prototype of spot-scanning arc treatment (SPArc) delivery was performed on a clinical proton beam therapy machine [30]. PAT has been shown in treatment planning studies to lower the dose to organs at risk compared to modern conformational techniques, such as intensity-modulated proton therapy and VMAT, for several cases and sites, such as pediatric cancers [31], adult brain tumors [9], breast [12] or lung [11], among others. An additional potential advantage of PAT is the possibility of performing a radiobiological optimization [13]. Some recent studies indicate that PAT might allow for LET painting, which can be used to increase the relative biological effectiveness within the target [31,32].

pMBRT is a novel experimental technique that has proven to significantly widen the therapeutic window for brain tumors in small animal experiments [19,21,33]. The biological mechanisms are not totally understood but effective immunomodulation seems to play a major role in the anti-tumor response to pMBRT [34]. Other possible participants are radiation-induced bystander effects and vascular alterations. Those studies used a single array of proton minibeams with very high peak doses (58 to 70 Gy) in one fraction. Treatment planning studies have shown that the spatial modulation of the dose in pMBRT can be maintained in depth even in the case of large-tumor irradiations [26].

In this work, we showed that pMBAT further increases the individual benefits of PAT and pMBRT. pMBAT has proven to maintain the spatial fractionation of the dose in the normal tissues. Thereby, the normal tissue preservation of pMBRT is expected to add up to the benefits of dose reduction in PAT [9,11,12,32], increasing further the therapeutic index. A quasi-homogeneous dose distribution was obtained within the PTV (PVDR ≈ 1.2). It should be highlighted that a homogeneous dose distribution is not requested in pMBRT for effective tumor control or even tumor ablation [16,17,18]. Indeed, a percentage of 67% long-term survivals free of tumors was obtained in glioma-bearing rats treated with pMBRT with PVDR of 1.2 in the tumor [17]. A higher level of homogenization can be obtained, if needed, by optimizing the different irradiation parameters. Additionally, for the same average dose to the target and independently of the configuration (number of arrays, arcs separation, etc.), pMBAT offers up to 90% reduction in peak and valley doses as compared with one-array pMBRT, sparing normal tissue, especially at shallow depths and the skin. These reductions in peak and valley doses are achieved at the expense of larger volumes being irradiated with lower doses. However, the integral dose remains the same for an organ such as the whole brain in our study.

pMBAT also results in lower valley doses as compared with PAT. This constitutes a potential advantage. In micro and minibeam radiation therapy, valley doses are considered to be the most relevant parameter and seem to have a proportional relation with normal tissue tolerances [35]. Moreover, contrary to FLASH therapy [36] the normal tissue preservation in MRT and MBRT does not seem to have a low dose threshold [37,38], even at 2 Gy valley dose [38]. This opens the door to the use of more aggressive irradiation schemes and hypofractionation, which could be useful in the management of difficult-to-treat cases. pMBAT also maintains the benefits of PAT in terms of the reduction in high-LET levels to normal tissues while adding the benefits of the spatial fractionation of the dose. As compared to pMBRT, pMBAT achieves a reduction in the volume exposed to higher LET levels.

In this proof-of-concept study, we also have shown that independently of the pMBAT plan configuration (i.e., number of arrays to create the arc, the separation between arrays or the PTV size), the dose to normal tissues is reduced as compared to pMBRT. Further evaluations considering more complex geometries and tumor types are needed to assess in depth the benefits of pMBAT. Finally, pMBAT could be implemented using technologies and protocols similar to those already implemented at our institution for pre-clinical trials in pMBRT [39].

## 5. Conclusions

This manuscript presents the proof-of-concept of a new treatment modality, called Proton Minibeam Arc Therapy (pMBAT). This study demonstrated that pMBAT not only maintains but significantly increases the individual benefits of pMBRT and PAT for normal tissue sparing. The same level of spatial modulation of the dose in normal tissues, as in single-array pMBRT irradiations, along with up to 90% reduction in both peak and valley doses may be achieved. pMBAT also reduces the high-dose and -LET normal tissue volumes as compared to pMBRT. These results can facilitate the translation of pMBRT to patients’ treatments by enabling with the use of lower peak doses than those used in the single array studies and further dose escalation in the tumor.

## Figures and Tables

**Table 1:** pMBAT plans description.

Case Number	Number of Arrays	Angular Separation	c-t-c	PTV Diameter
Number of arrays				
1	13	15°	4000 μm	20 mm
2	11	15°	4000 μm	20 mm
3	9	15°	4000 μm	20 mm
4	7	15°	4000 μm	20 mm
Angular separation				
5	9	20°	4000 μm	20 mm
6	9	15°	4000 μm	20 mm
7	9	10°	4000 μm	20 mm
c-t-c distance				
8	13	15°	4000 μm	20 mm
9	13	15°	2800 μm	20 mm
10	13	15°	2000 μm	20 mm
PTV size				
11	13	15°	4000 μm	20 mm
12	13	15°	4000 μm	30 mm

**Table 2:** Integral doses on the PTV and brain volume.

	Integral Doses (a.u.)	
	PTV	Brain
PAT	1.00 ± 0.04	0.30 ± 0.02
pMBRT	1.00 ± 0.05	0.32 ± 0.03
pMBAT	1.00 ± 0.05	0.33 ± 0.03

**Table 3:** Mean LET_d_ in the PTV and brain volume.

	Mean LET_d_ (keV/μm)	
	PTV	Brain
PAT	3.4 ± 0.2	1.7 ± 0.1
pMBRT	3.5 ± 0.2	2.0 ± 0.1
pMBAT	3.6 ± 0.2	2.0 ± 0.1

## Data Availability

The data presented in this study are available on request from the corresponding author.

## Supplementary Materials

The following are available online at https://www.mdpi.com/article/10.3390/cancers14010116/s1, Figure S1: Depth dose profiles, Figure S2: Coronal view of the pMBAT dose distributions, Figure S3: Coronal view of the dose distributions in the pMBAT scenario, Table S1: PVDR values as a function of depth.
