# Supplementary material for: Proton Minibeam Radiation Therapy and Arc Therapy: Proof of Concept of a Winning Alliance
Source: Cancers (Basel). 2021 Dec 27;14(1):116. doi: 10.3390/cancers14010116 (PMC8749801; doi:10.3390/cancers14010116)
Supplement: Supplementary file 1 [file cancers-14-00116-s001.zip › cancers-1502811-SI.pdf]

Supplementary Materials: Proton Minibeam Radiation Therapy and Arc Therapy: Proof of Concept of a Winning Alliance

Ramon Ortiz, Ludovic De Marzi and Yolanda Prezado

Table S1. PVDR values in normal tissues as a function of depth in the standard pMBRT (single-array) and pMBAT (multi-array) scenarios.

Depth (cm)	PVDR	
	pMBRT	pMBAT
0	12.2 ± 0.6	12.2 ± 0.6
1	9.0 ± 0.5	9.2 ± 0.5
2	6.1 ± 0.4	6.3 ± 0.4
3	4.8 ± 0.3	4.6 ± 0.3
4	3.3 ± 0.2	2.7 ± 0.2
5	2.1 ± 0.1	1.9 ± 0.1
6	1.65 ± 0.08	1.47 ± 0.07
7	1.43 ± 0.07	1.30 ± 0.06

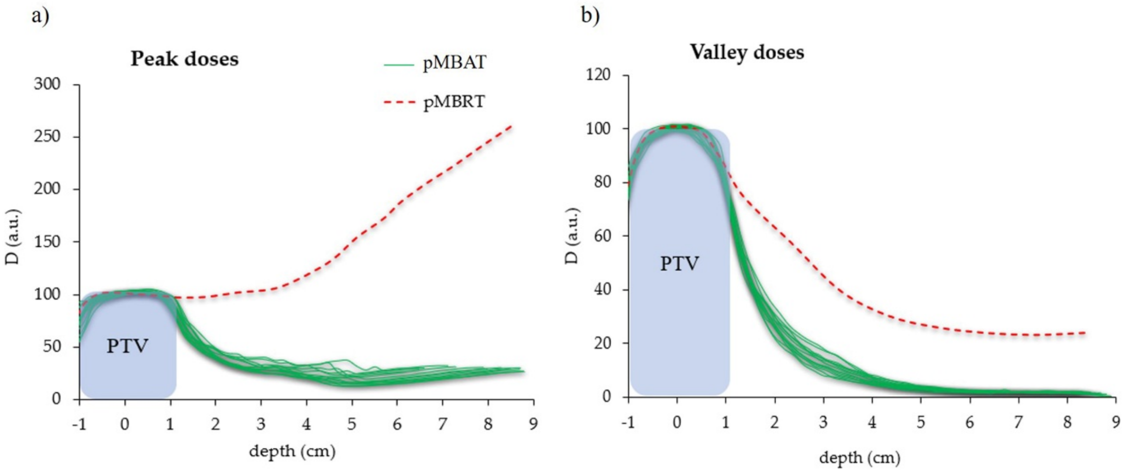

Figure S1. Depth dose profiles at (a) peak and (b) valleys positions of the standard pMBRT (single-array) and pMBAT (multi-array) scenarios. The 13 arrays composing the pMBAT are presented. The origin of the axis of abscissas (depth) is at the PTV position. Doses are normalized to the maximum dose at peak and valleys, respectively.

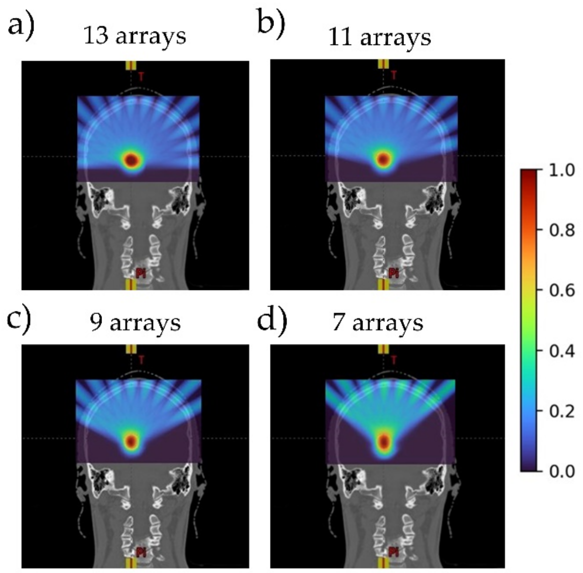

Figure S2. Coronal view of the pMBAT dose distributions. Three plans consisting of 13, 11, 9 and 7 arrays are presented.

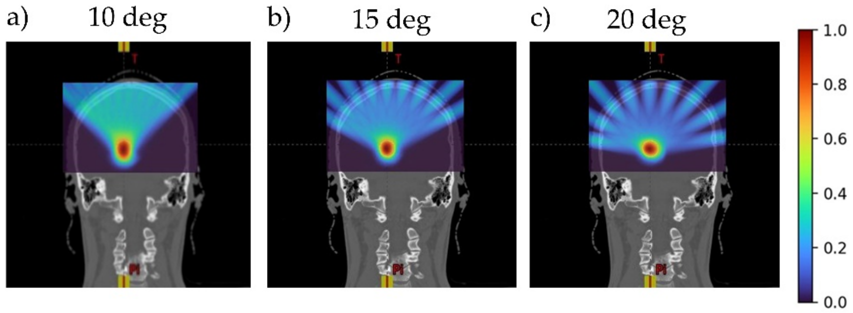

Figure S3. Coronal view of the dose distributions in the pMBAT scenario. Three plans considering a different angle separation between the fields that form the arc (10, 15 and 20 degrees) are presented.
